# Whole transcriptome sequencing and ceRNA regulation network profiling of heat acclimation in protecting against heat stress injury in rat myocardium

**DOI:** 10.1371/journal.pone.0348180

**Published:** 2026-04-29

**Authors:** LiJun Fan, JiaJun Chen, QingHan Zhang, MingXiao Song, ZiYi Guo, XueWei Chen, JunYu Lu, Jing Wang

**Affiliations:** Military Medical Sciences Academy, Tianjin, China; Xiangya Hospital Central South University, CHINA

## Abstract

Heat acclimation (HA) has emerged as a proven protective intervention to augment thermotolerance and mitigate heat stress (HS)-induced myocardial injury. Despite its clinical significance, the regulatory roles of noncoding RNAs associated with HA-mediated cardioprotection remain largely unexplored. This study conducted comprehensive transcriptomic profiling to delineate the molecular mechanisms underlying HA-induced cardioprotection against HS. Using well-characterized rat models of HS and HA preconditioning, we performed high-throughput sequencing on myocardial tissues to map circRNA/lncRNA expression landscapes. Bioinformatic analyses were integrated with functional validation to identify key regulatory elements. HA preconditioning markedly attenuated HS-induced injury, reducing inflammatory cytokines (IL-1β ↓ 24.36%, IL-4 ↓ 11.3%) while elevating IL-6 (+25.67%), suggesting immunomodulatory rewiring. HS leads to elevated serum HSP70 levels. Analysis identified nine core molecular candidates—miR-196c-5p, miR-212-3p, miR-212-5p, *Rffl, Rassf1, Ppp3cc, Zbtb20, Cdh5,* and *Cxcl2*—and delineated two central ceRNA regulatory axes: MSTRG.6276.3–miR-196c-5p–*Rffl* and MSTRG.4016.1–miR-122-5p–*Rassf*1. These networks potentially coordinate mitochondrial integrity, hypoxic adaptation, and apoptotic regulation through modulation of inflammatory signaling, metabolic homeostasis, and calcium pathways. The findings establish a noncoding RNA-defined regulatory framework for HA-mediated cardioprotection, revealing novel therapeutic targets for cardiovascular disorders triggered by thermal stress.

## 1. Introduction

Acute or chronic exposure to high temperature environments, can induce heat stress (HS). This condition involves the activation of multiple physiological effectors, including changes in working efficacy, core temperature, heart rate, and neuroendocrine responses, and may even progress to heat stroke, which can trigger a cascade of critical pathological changes such as systemic inflammatory response syndrome, shock, and multi-organ dysfunction syndrome [1]. The heart is the most temperature-sensitive organ, and HS can elicit morphological, metabolic, and biochemical alterations in the myocardium, and lead to long-term cardiovascular dysfunction [2, 3]. The influence of heat exposure on adverse cardiovascular health outcomes has been widely reported [4, 5]. According to the American Heart Association, the relative risks of cardiovascular disease-related deaths at high ambient temperatures have remarkably increased, leading to doubling to tripling of mortality [6]. Previous work also suggests that high temperatures aggravate the burden of cardiovascular disease [7]. Consequently, the cardiovascular effects induced by HS have garnered considerable attention.

Currently, heat acclimation (HA) is regarded as one of the most effective measures to enhance the heat-tolerance ability of individuals, thereby slowing down and preventing the occurrence of heat injury. HA can significantly enhance thermoregulation, and heat tolerance, in organism while concurrently reducing the incidence of heat-related ailments such as heat cramps, heat exhaustion, heat syncope, and severe heat stroke [8, 9]. HA also exerts beneficial effects on cardiovascular stability. It enhances cardiovascular stability by increasing plasma volume and reducing heart rate, while also mitigating heat stress-induced cardiovascular damage through modulation of vascular endothelial function [9, 10]. The protective capacity of HA against HS has also been documented, heat preconditioning is a critical factor in safeguarding against hepatic and brain injury resulting from HS in rodent [11, 12]. However, the mechanism by which HA protects the cardiovascular system from HS-induced injury remains unclear. Thus, it is necessary to investigate the specific targets and molecular mechanisms underlying the protective effects of HA in preventing HS in the myocardium.

Elucidation at the transcriptional level is crucial for comprehending the adaptive regulation of HA protection against HS. Noncoding RNA, encompassing circRNA, lncRNA, miRNA, and other functional RNAs play pivotal roles in numerous physiological and pathological processes, such as cell growth, embryogenesis, diseases, gene regulation, signal transduction, and receptor activation [13]. In the myocardium, these non-coding RNAs and their target mRNAs exhibit cell-type-specific distribution, localizing predominantly to cardiomyocytes and vascular endothelial cells. Study have demonstrated that the activation of YAP/TAZ induces HS transcriptome and cell survival, and plays a crucial role in facilitating cellular adaptation to HS [14]. The intensity and duration of HS may impact the activation of specific microRNAs. miR-125b, miR-154, and miR-382 are activated during mild HS [15], whereas miR-155 is activated during high HS to promote HS-induced inflammatory responses [16]. Furthermore, NC-001782, APOA4, and APOA5 have been proposed to mitigate HS-induced damage through their synergistic action on the transcriptome in Hu sheep liver tissues [17]. Nonetheless, there is a paucity of studies on the expression profiles of myocardial transcription characteristics by HA and the effect of HA against HS.

In this study, we scrutinized the safeguarding impact and mechanism of HA against HS using whole-transcriptome sequencing, and established circRNA/lncRNA-associated competing endogenous RNA (ceRNA) regulatory networks. Our findings reveal the distinctive expression profile of the protective effect of HA against HS and its associated regulatory pathways, thereby serving as a valuable resource for comprehending the intrinsic regulatory molecular mechanism and potential biomarkers of the protective effects of HA against HS in rat myocardium.

## 2. Materials and methods

### 2.1. Animals and experimental conditions

Animal experiments were conducted in compliance with the National Institutes of Health guide for the care and use of Laboratory animals (NIH Publications No. 8023, revised 1978) and authorized by the Ethics Committee for Animal Experimentation of the Military Medical Sciences Academy (IACUC of AMMS-04-2021-017). A total of 46 male Sprague Dawley rats (8weeks, 180–200 g) were acclimatized for one week under controlled environmental conditions, including an ambient temperature of 23°C, relative humidity (RH) of 50%, and a 12h/12h light–dark cycle (lights on at 6:00 am). All rats were provided access to food and water ad libitum. The rats were randomly assigned to one of the following four groups; Control (C) group, Heat Acclimation (HA) group, Heat Stress (HS) group, and Heat Acclimation Pre-condition Heat Stress (HA + HS) group. Rats in the control group(n = 12) were housed in a chamber maintained at a temperature of 23 ± 0.5°C and provided access to food ad libitum for 30 days. Rats in the HA group(n = 12) were subjected to specific conditions (hot chamber, 35 ± 0.5°C; RH, 60% ± 5%; 2 h daily for 30 days) that induced HA homeostasis, as evidenced by the growth rate of body weight and rectal temperature (Tre) [18–20]. Rats in the HA + HS(n = 12) and HS groups(n = 10) were subjected to hot conditions in a chamber (42 ± 0.5°C; RH, 60% ± 5%; 30 min daily for 3 days). Rats in the C and HA groups had their body weights and food intake measured at a fixed time daily, whereas Tre was measured every 10 days before and after HA using an animal heat simulation module (product model: DWX-400FPY; Beijing Luxi Co. Ltd, China) and a thermal probe (SN2016028; Shandong Co. Ltd, China) inserted 5 cm into the rectum. The Tre and body weights of mice in the HS and HA + HS groups were measured before and after exposure to each heat-stress session. Rats were subjected to profound anesthesia using sodium pentobarbital (100 mg/kg, intraperitoneally) prior to being exsanguinated through the abdominal aorta and subsequently euthanized via cervical dislocation for the purpose of myocardial tissue collection. Animals that met predetermined humane endpoints, characterized by immobility, loss of righting reflex, or severe convulsions, were promptly euthanized with an overdose of sodium pentobarbital (150 mg/kg, intraperitoneally) to mitigate suffering. All experiments were conducted in accordance with the Animal Protection Law of China and ethical guidelines for animal treatment.

### 2.2. ELISA to determine the levels of heat shock protein, oxidative stress, and inflammatory factors

Blood samples were left to stand at room temperature for 2 h, centrifuged at 1000 × g for 20 min, and the supernatant was aliquoted and stored at −20°C. Serum heat shock protein 70 (HSP70, JL11282-96T), serum malondialdehyde (MDA, JL13297-96T), serum superoxide dismutase (SOD, JL22893-96T), serum glutathione peroxidase (GSH-px, JL21016-96T), serum interleukin-1β(IL-1β, JL20884-96T), serum interleukin-4 (IL-4, JL20894-96T) and serum interleukin-6 (IL-6, JL20896-96T) levels were measured using commercially available rat ELISA kits (Shanghai Jianglai Biotechnology Co., Ltd., China) and following the manufacturer’s instructions. Absorbance at 450 nm was measured using a microplate reader (RT-6100, Rayto, China), and concentrations were determined using ELISACalc software (Cornple-Software, Iowa City, IA, USA).

### 2.3. Hematoxylin and Eosin staining

Histopathological analysis was performed on 3 μm cardiac tissue sections stained with hematoxylin and eosin (C0105S, Beyotime), following the protocol provided by the manufacturer.

### 2.4. RNA isolation, library preparation, and sequencing

Total RNA from the myocardium left ventricular of rats was extracted using TRIzol reagent (Invitrogen, NY, USA). Prior to sequencing, RNA samples were assessed for both quantitative and qualitative integrity using Qubit 4 Fluorometer (Thermo Fisher Scientific, lnc.) (Concentration ≥ 300 ng/μl; OD260/OD280 ≥ 2.0) and by performing 1% agarose electrophoresis. The purified RNA was subsequently stored at –80°C. RNA samples were then submitted to Jenergy Biotechnology Shanghai Co., Ltd (China) for RNA sequencing. Paired-end sequencing was conducted utilizing the Illumina NovaSeq 6000 platform, employing the NovaSeq 6000 S4 reagent kit (PE150, Illumina, USA), in accordance with the protocols specified by the manufacturer. The study employed high-throughput sequencing of numerous samples, utilizing an insert size of 300–450 bp to generate the ultimate cDNA library. Additionally, the miRNA library was prepared using Illumina’s TruSeq® small RNA Sample Prep Kit v2 (RS-200–0012) and subjected to single terminal 50 bp sequencing mode on the Illumina HiSeq 6000 high-throughput sequencing platform.

The fragments per kilobase of transcript per million reads mapped value was used to determine mRNA and lncRNA expression levels, the back spliced reads per billion mapping reads value was utilized to quantify circRNAs, and the counts per million metrics was used to measure miRNA abundance. Mirdeep2 (v2.0.0.5, https://www.osc.edu/book/export/html/4389) and miRanda (v3.3a, http://www.microrna.org/microrna/getDownloads.do) software were used for the prediction of novel miRNAs and target genes, respectively. Differential expression analysis was conducted using DESeq2(v1.16.1), with screening conditions uniformly set to |log2(fold change)| ≥ 1.0 and *P* < 0.05 to determine significant differential expression. Then, a cluster analysis is conducted, DEGs underwent Z-score normalization across the samples. Subsequently, hierarchical clustering was conducted utilizing Euclidean distance and complete linkage to categorize genes according to their expression profiles. The clusters obtained from this analysis were employed to identify co-expressed gene modules.

### 2.5. Gene Ontology (GO) and Kyoto Encyclopedia of Genes and Genomes (KEGG) functional enrichment and pathway analysis

GO biological function enrichment and KEGG signaling pathway enrichment were performed to elucidate the role of differentially expressed mRNAs and the potential target genes of lncRNAs, circRNAs, and miRNAs in the context of HA against HS. GO enrichment analysis was conducted using TopGO software (Release 3.21), and KEGG pathway enrichment analysis was performed using KEGG Pathway (Release 104.0). For both analyses, all genes were used as the background list, and the target gene list was defined as the candidate list screened from the background. *P*-value were adjusted using Fisher’s exact test, followed by FDR correction using the Benjamini & Hochberg method for multiple testing. tatistical significance was set at *P* < 0.05.

### 2.6. RT-qPCR validation

Total RNA was extracted from the myocardium of rats, as previously described, and SuperScriptTM IV First-Strand Synthesis System and Powerup^TM^ SYBR^TM^ Green Master Mix were used (Invitrogen Trading Co.Ltd., Shanghai, China), following the manufacturer’s instructions. cDNA was subjected to amplification for miRNA RT-qPCR using the Applied Biosystems ViiA 7 Dx real-time PCR system (4453535, Thermo Fisher Scientific, lnc.) in accordance with the manufacturer’s instructions. GAPDH (circRNA, lncRNA, and mRNA) and U6(miRNA) were used as normalization controls. Relative expression levels were calculated using the 2^-ΔΔCT^ method. A total of 6 circRNAs, 7 lncRNAs, 5 miRNAs, and 10 mRNAs were verified based on three criteria, namely, commonality among the four groups, highest expression multiple change, and literature analysis. For qPCR validation, each group contained 6–12 biological replicates. The primers are listed in Table 1.

**Table 1:** Primers for RT-qPCR.

Gene	Forward primer	Reverse primer
***Gapdh***	CAGTGCCAGCCTCGTCTCAT	AGGGGCCATCCACAGTCTTC
***U6***	GCTTCGGCAGCACATATACTAAAAT	CGCTTCACGAATTTGCGTGTCAT
**1:163203870|163216364**	AGAGCCTATATTCCATCAGGGA	TCTAAAGCCTTGAATTGCACCA
**4:58469325|58485447**	TGGGAGTCTTGCAGCCTCTC	TCCCTGGGATGCAATGGTGG
**X:68338536|68352920**	GACAGAAGGGTTGTACCGCA	TGGTGAGTGTCCTGGTCTCT
**3:63717453|63748423**	AGAAGACTGCCGTTCCTGTG	TTTCGGATGAAGGACGGTGG
**7:127100247|127104943**	CTGACACTCATGCCCTTGGT	GTGCTGGGCTCCATACACAT
**6:52131586|52143735**	ATTCTGTTCCAGCGGCAGAG	CTGTCTTCTTTTCACGGCATTCA
**MSTRG.4016.1**	ATCAGTGCTGACAGACCTGC	GGATGGGAGAGAGGGGAACT
**MSTRG.13563.7**	TGAAGGGAGGGAGAGATGGG	AGGGACCATAAGCAGGCCTA
**MSTRG.7374.5**	ATAGCAATGGTGGCTGCCAA	TCCCAGAGCAGAGACCTTCA
**MSTRG.4918.2**	TCACACCAGACTTTTCGCCA	CTGGGCTGGCATTTGTGTTC
**MSTRG.14793.4**	TGAGATGACTGCAGCAGTGG	GGTATGGGTGTTGGGAGTGG
**MSTRG.15354.1**	AGGGAAGAGCGAGAAAACGG	GAGTGTTCCTCCTTCTCGCC
**MSTRG.6276.3**	GGCTATGGTTGTGGCTCTGT	CACAAGTGTCTGTGCTGTGC
**miR-122-5p**	ACAGCGCTGGAGTGTGACAATGGTGT	GTGCGTGTCGTGGAGTCG
**miR-196c-5p**	GCGCACGCGTAGGTAGTTTCGTGTTG	GTGCGTGTCGTGGAGTCG
**miR-1949**	CGCAGCGCTATACCAGGATGTCAGCA	GTGCGTGTCGTGGAGTCG
**miR-212-3p**	GCGTAACAGTCTCCAGTCACGGCCA	GTGCGTGTCGTGGAGTCG
**miR-212-5p**	ACCTTGGCTCTAGACTGCT	GCAGGGTCCGAGGTATTC
***Rffl***	ACTCAGCAGTCCGTTGACTC	ACGGTCAGGCCTTCAATGTC
***Rassfl***	CGTGAAGGTCCTCCTCTGATG	CGCGCCATATCCACTGGTAG
***Ghr***	ATAGTGCAACCTGATCCGCC	TCCCTTCAGAACATCGGCAC
***Zbtb20***	GTGCTGAGAGTCTCCCAGTCG	GAATCCTGGATGCCTGGGAAC
***Them4***	GCTGCAGGAACTCACTCTCA	GCAGAGCAGGTCCTATGCTT
***Chd5***	GCCAGAGCCAGTTGGAAAAC	TCTTCAGTCTGTCGAGGGCT
***Erbb2***	GAGTACCATGCAGATGGGGG	TCCATCGTAAGGTTTGGCCC
***Cxcl2***	TCCCTCCTGTGCTCAAGACT	ACCCCTGTACCCTGATGGTT
***Ppp3cc***	CGAGCGCGTCATCAAAGCTG	TACGTCCTCTTCTACCCGGC
***Ms4a6a***	AGTGGGGCAAATGGGTATCA	CATGCCACATGCACGGATTA

### 2.7. ceRNA network construction

The phenomenon of gene silencing can be attributed to the binding of miRNAs to mRNA, whereas the presence of ceRNAs can serve as a mechanism to impede miRNA binding and counteract its repressive effects, ultimately leading to the formation of a ceRNA network. MiRanda (v3.3a) was used to predict the targets of differentially expressed miRNAs (Tot Score ≥ 150, Tot Energy ≤ –20), resulting in the construction of network maps depicting the relationship between miRNAs and their target genes. MiRanda (v3.3a) was also to predict the miRNA binding sites within various circRNA sequences, leading to the creation of a global network illustrating the relationship between miRNAs and target circRNAs (Tot Score ≥ 150, Tot Energy ≤ –20). The ceRNA network was constructed using Cytoscape, and the analytical method by Chu et al. was used to construct the ceRNA network [21].

### 2.8. Statistical analysis

Statistical analyses were conducted utilizing SPSS Statistics (v22.0). The data are expressed as the means ± s.e.m. Visualization was accomplished using GraphPad Prism (v9.0), Figdraw (v2.0), and Sangerbox (v3.0). For comparisons between two groups, an independent samples t-test was employed, whereas one-way ANOVA was utilized for analyses involving multiple groups. In the case of two-factor designs, two-way ANOVA was performed to assess the main effects of each factor and their interaction. When a statistically significant interaction was identified (*P* < 0.05), further simple effects analysis was conducted to investigate the influence of one factor at each level of the other. For post hoc multiple comparisons, the selection of the test was contingent upon the homogeneity of variance: the LSD test was applied when variances were equal, while Dunnett’s T3 test was used when variances were unequal, with *P* < 0.05 indicating statistical significance.

## 3. Results

### 3.1. Heat defense responses during HA/HS

**Fig 1:**
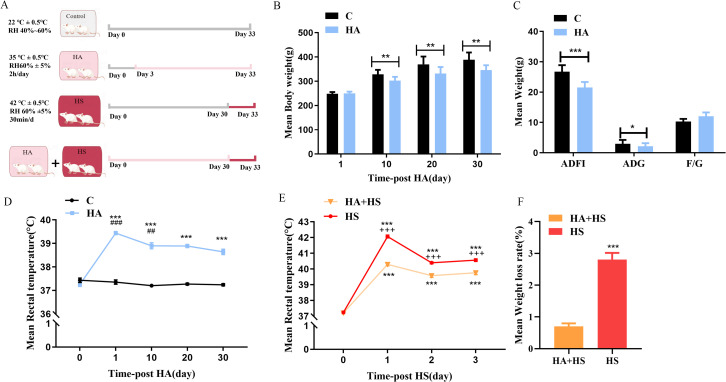
The impact of heat acclimation (HA) and heat stress (HS) on rat models. **(A)** Heat acclimation and heat stress rat models establish condition. Gray, pink, and dark red indicate room temperature without intervention, HA, and HS, respectively. **(B)** The HA group exhibited a significantly lower body weight compared to the C group (** *P* < 0.01). **(C)** HA’s effect on rats’ growth performance (Average daily feed intake, ADFI *** *P* < 0.001, Average daily gain, ADG * *P* < 0.05). **(D)** Rectal temperature measurements revealed significant differences between the HA and C groups on days 1 to 30 following heat exposure (1d, 10d, 20d, 30d *** *P* < 0.001), as well as on days 0 and 10 for the HA group alone (0d vs 1d ###*P* < 0.001, 1d vs 10d ## *P* < 0.01). **(E)** Rectal temperature changes were evaluated in the HA + HS and HS groups (Each day compared with the same group before heat exposure *** *P* < 0.001, each day compared with the HS group +++ *P* < 0.001). **(F)** The weight loss rate in the HA + HS compared with the HS group (***P < 0.001).

A hot chamber was utilized to establish a distinct heat-exposed rat model to investigate differential gene expression after subjecting to HS (Fig 1A). Compared to normothermic controls, HA rats exhibited modestly reduced weight gain over 10, 20, and 30 days (*P* < 0.01; Fig 1B), which was associated with decreased average daily gain (ADG) and average daily feed intake (ADFI) (*P* < 0.05; Fig 1C). This growth modulation appeared to be driven primarily by reduced ADFI, as feed efficiency (F/G) remained unchanged. Tre was significantly elevated in HA rats at days 1, 10, 20, and 30 relative to controls (*P* < 0.001), before gradually declining and stabilizing beyond day 10 (Fig 1D)—consistent with established adaptive responses to HA [22–24]. These data confirm the successful induction of the HA state in our model. Notably, HA preconditioning conferred significant protection against subsequent HS challenge: HA rats exposed to HS showed a markedly lower Tre (*P* < 0.01; Fig 1E) and weight loss (*P* < 0.01; Fig 1F) compared to rats subjected to HS alone.

Heat acclimation triggers a coordinated adaptive response that stabilizes core temperature and limits metabolic imbalance during prolonged heat exposure. Furthermore, HA preconditioning confers robust thermotolerance and preserves body weight under acute HS, highlighting its potential as an effective intervention to mitigate HS-induced physiological disruption.

### 3.2. Impact of HA/HS on stress and inflammatory factor responses in rat serum

**Fig 2:**
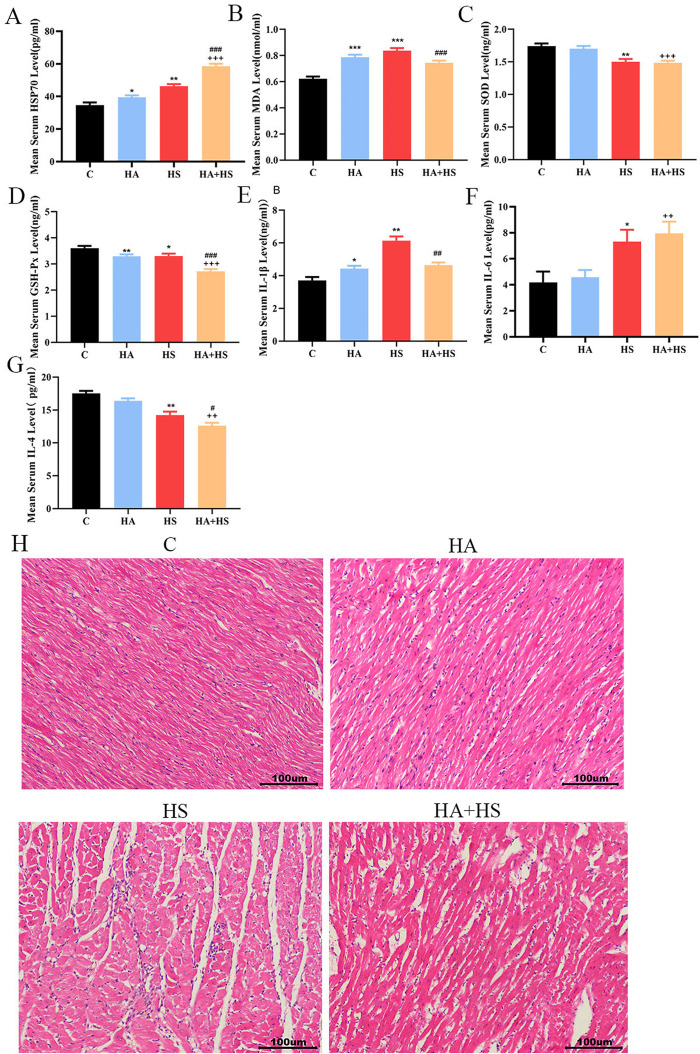
The impact of HA on HS-induced stress and inflammatory factors in rat serum. **(A)** Levels of HSP70 in rat serum. **(B)** Levels of SOD in rat serum. **(C)** Levels of MDA in rat serum. **(D)** Levels of GSH-px in rat serum. **(E)** Levels of IL-1β in rat serum. **(F)** Levels of IL-6 in rat serum. **(G)** Levels of IL-4 in rat serum. **(H)** HE staining of myocardial tissue. n = 3, Scale bar: 100 μm. * Compared with C group; # compared with HS group; + compared with HA group. C group, n = 11; HA group, n = 11; HA + HS group, n = 12; HS group, n = 10) *P < 0.05, ** *P* < 0.01, *** *P* < 0.001; # *P* < 0.05, ##*P* < 0.01, ### *P* < 0.001; + P < 0.05, ++ *P* < 0.01, +++ *P* < 0.001.

The molecular and physiological alterations underlying HA-mediated protection were investigated. Analysis of the classic thermotolerance chaperone HSP70 revealed its significant induction in both HA and HS rats compared to controls. Strikingly, HA preconditioning prior to HS challenge further enhanced HSP70 accumulation beyond levels observed in HA or HS alone (Fig 2A), suggesting a synergistic induction that may contribute to enhanced proteostasis under severe stress.

Key redox markers were measured to assess oxidative stress status. Serum MDA levels, an indicator of lipid peroxidation, were elevated in HA and HS groups relative to controls. However, HA preconditioning modestly attenuated this increase upon HS challenge (Fig 2B). Concurrently, antioxidant capacity was compromised by HS, as shown by a pronounced decrease in SOD activity, which was partially rescued by HA preconditioning. In contrast, GSH-px activity was significantly suppressed in both HA and HS conditions and further reduced in HA-preconditioned HS rats (Fig 2C and D). These data indicate that HA remodels the antioxidant defense system, enhancing certain free radical–scavenging activities while permitting a selective reduction in peroxidase capacity, potentially as part of a coordinated adaptive response.

We further evaluated the inflammatory response. Pro-inflammatory cytokines IL-1β and IL-6 were elevated following HS, whereas HA preconditioning significantly blunted the IL-1β response (Fig 2E). IL-6 remained highly induced by HS even after HA preconditioning (Fig 2F), indicating its distinct regulation. Conversely, the anti-inflammatory cytokine IL-4 was markedly reduced by HS, a response not reversed by HA preconditioning (Fig 2G). This suggests that HA selectively modulates specific inflammatory pathways, preferentially restraining certain pro-inflammatory mediators rather than broadly suppressing inflammation.

HA remodels the cellular stress response by potentiating HSP70 expression and inducing a reconfigured antioxidant defense system, leading to attenuated lipid peroxidation and enhanced free radical clearance under HS. Furthermore, HA preconditioning selectively restrains the production of specific pro-inflammatory cytokines such as IL-1β, but does not fully reverse the anti-inflammatory deficit or all inflammatory perturbations induced by HS. Thus, HA confers a multi-faceted protective adaptation that enhances proteostasis, rebalances redox status, and tempers specific arms of the inflammatory response.

The myocardial pathological staining results are presented in Fig 2H. In both the C and HA groups, the myocardial tissue structure appeared predominantly normal, characterized by orderly cardiomyocyte arrangement and clearly discernible striations. Conversely, the HS group demonstrated widened intercellular spaces accompanied by inflammatory cell infiltration. Relative to the HS group, the HA + HS group exhibited a marked reduction in inflammatory cell infiltration, with only sporadic widening of the intercellular spaces observed. It indicates that HS caused a certain degree of myocardial damage, while HA effectively protected against the myocardial damage mediated by HS.

### 3.3 Identification of differentially expressed lncRNAs, circRNAs, mRNAs and miRNAs by sequencing

**Fig 3:**
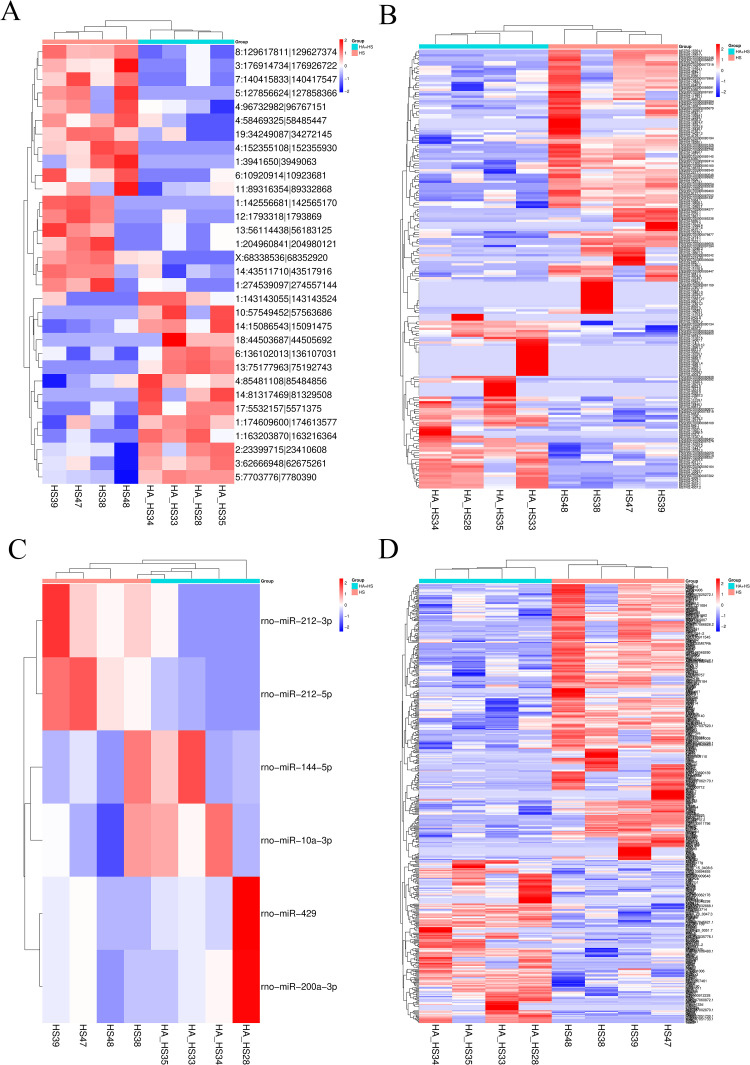
Heatmaps depicting the expression of circRNAs(A), miRNAs (B), lncRNAs (C), and mRNAs (D). Heatmap plot of DEcircRNAs, DEmiRNAs, DElncRNAs and DEmRNAs expression profiles between HS vs HA + HS with |log2(fold change)| ≥ 1.0, *P* < 0.05. Expression values are represented in accordance with the color scale, where red denotes upregulation and blue denotes downregulation. Each column corresponds to a single sample, and each row pertains to a transcript.

Whole-transcriptomic analysis represents a highly effective method for determining the biological role of RNAs in rat myocardium with respect to the protective effects of HA against HS injury. By applying the screening criteria (|log2(fold change)| ≥ 1 and *P* < 0.05), the differentially expressed RNAs were identified through sequencing in various contrast groups (Table 2). Unsupervised hierarchical clustering of these transcriptional profiles revealed a clear and pronounced separation between HA-preconditioned rats subsequently exposed to HS and those subjected to HS alone (Fig 3). This distinct global expression pattern signifies that HA preconditioning drives a fundamental reprogramming of the myocardial transcriptome, establishing a unique molecular landscape that underpins the acquired tolerance to severe HS.

**Table 2:** Differentially expressed (DE) RNAs in different contrast group.

Contrast group	DEmRNAs	DEmiRNAs	DEcircRNAs	DElncRNAs
**C vs HA**	1294	12	43	242
**C vs HS**	749	15	38	178
**HA vs HA + HS**	1338	20	44	360
**HS vs HA + HS**	837	6	32	191

### 3.4. Comparative pathway analysis of significant mRNAs in rat myocardium

Functional enrichment analysis of differentially expressed mRNAs, informed by lncRNA and circRNA profiles, reveals that heat acclimation (HA) confers cardioprotection through a coordinated transcriptional pre-adaptation (Table 3 and Table 4). GO term analysis demonstrates that HA preconditioning (C vs HA) fundamentally rewires cardiac metabolism by enhancing mitochondrial integrity, respiratory chain assembly, and ATP synthase activity. In stark contrast, acute heat stress (C vs HS) induces a transcriptomic signature of sterile inflammation, dominated by MHC class II-mediated antigen presentation. Critically, the protected state in HA-preconditioned hearts challenged with HS (HS vs HA + HS) is defined by a distinct transcriptional shift. This shift is characterized by a modulated immune response alongside sustained energy homeostasis, indicating that HA establishes a new immunometabolic set-point rather than simply suppressing the stress response.

**Table 3:** The top 3 GO enriched analysis of DEmRNAs in different contrast group.

Contrast group	GO_category		
	Cellular component	Biological process	Molecular function
**C vs HA**	mitochondrial intermembrane space protein transporter complex	ATP metabolic process	3-phosphoinositide-dependent protein kinase binding
	mitochondrial proton-transporting ATP synthase complex, coupling factor F(o)	inner mitochondrial membrane organization	DNA translocase activity
	proton-transporting ATP synthase complex, coupling factor F(o)	NADH dehydrogenase complex assembly	primary active transmembrane transporter activity
**C vs HS**	plasma membrane part	antigen processing and presentation of polysaccharide and exogenous peptide antigen via MHC class II	protein binding
	MHC class II protein complex	polysaccharide assembly with MHC class II protein complex	tropomyosin binding
	early phagosome	negative regulation of MyD88-dependent toll-like receptor signaling pathway	peptide antigen binding
**HS vs HA + HS**	early phagosome	MHC class II protein complex assembly	ATPase activity
	MHC class II protein complex	peptide antigen assembly with MHC class II protein complex	protein binding
	MHC protein complex	antigen processing and presentation of peptide or polysaccharide and exogenous peptide antigen via MHC class II	peptide binding

**Table 4:** The top 3 KEGG pathways classified in each contrast group.

Contrast group	Pathway class（percentage of 37 pathways）	Pathway name	Rich factor
**C vs HA**	Nervous system (16.22%)	Retrograde endocannabinoid signaling	0.14
		Synaptic vesicle cycle	0.16
		Dopaminergic synapse	0.13
		Glutamatergic synapse	0.11
	Signal transduction (10.81%)	Calcium signaling pathway	0.11
		cGMP - PKG signaling pathway	0.11
		ErbB signaling pathway	0.12
		HIF-1 signaling pathway	0.11
	Neurodegenerative diseases (8.11%)	Huntingtons disease	0.18
		Parkinsons disease	0.18
	**Pathway class（percentage of 57 pathways）**	**Pathway name**	**Rich factor**
**C vs HS**	Infectious diseases (14.04%)	Staphylococcus aureus infection	0.25
		Leishmaniasis	0.18
		HTLV-I infection	0.07
	Immune diseases (12.28%)	Autoimmune thyroid disease	0.17
		Inflammatory bowel disease (IBD)	0.16
		Systemic lupus erythematosus	0.11
	Immune system (10.53%)	Th1 and Th2 cell differentiation	0.14
		Antigen processing and presentation	0.15
		Th17 cell differentiation	0.12
		Intestinal immune network for IgA production	0.17
		Platelet activation	0.07
	**Pathway class（percentage of 47 pathways）**	**Pathway name**	**Rich factor**
**HS vs HA + HS**	Infectious diseases (17.02%)	Staphylococcus aureus infection	0.23
		Kaposis sarcoma-associated cherpesvirus infection	0.07
		Herpes simplex infection	0.11
	Carbohydrate metabolism (12.77%)	Citrate cycle (TCA cycle)	0.70
		Propanoate metabolism	0.60
		Pyruvate metabolism	0.45
		Glyoxylate and dicarboxylate metabolism	0.46
		Glycolysis/ Gluconeogenesis	0.23
		Butanoate metabolism	0.28
	Immune system (10.64%)	Antigen processing and presentation	0.15
		Natural killer cell mediated cytotoxicity	0.11
		Th17 cell differentiation	0.13
		Hematopoietic cell lineage	0.13
		Th1 and Th2 cell differentiation	0.13

KEGG pathway analysis further contextualizes this rewiring. HA preconditioning enriches neuro-signaling and signal transduction pathways (e.g., retrograde endocannabinoid and calcium signaling), suggesting involvement of neural circuits in stress anticipation. The transition to the protected state (HS vs HA + HS) is marked by a fundamental pivot: a suppression of broad inflammatory pathways coupled with a robust enrichment of core energy metabolism pathways, including the TCA cycle and glycolysis.

It indicated that HA initiates a proactive transcriptional program that establishes a protected cardiac phenotype. This phenotype is primed for metabolic defense through enhanced mitochondrial efficiency and is immunologically recalibrated to mitigate maladaptive inflammation. Upon lethal heat stress, this pre-adapted state enables a critical metabolic prioritization, ensuring energy production and survival. Thus, HA-mediated protection hinges on a sophisticated, pre-programmed switch from an inflammatory toward a resilient metabolically fortified state.

### 3.5. Validation using RT-qPCR

**Fig 4:**
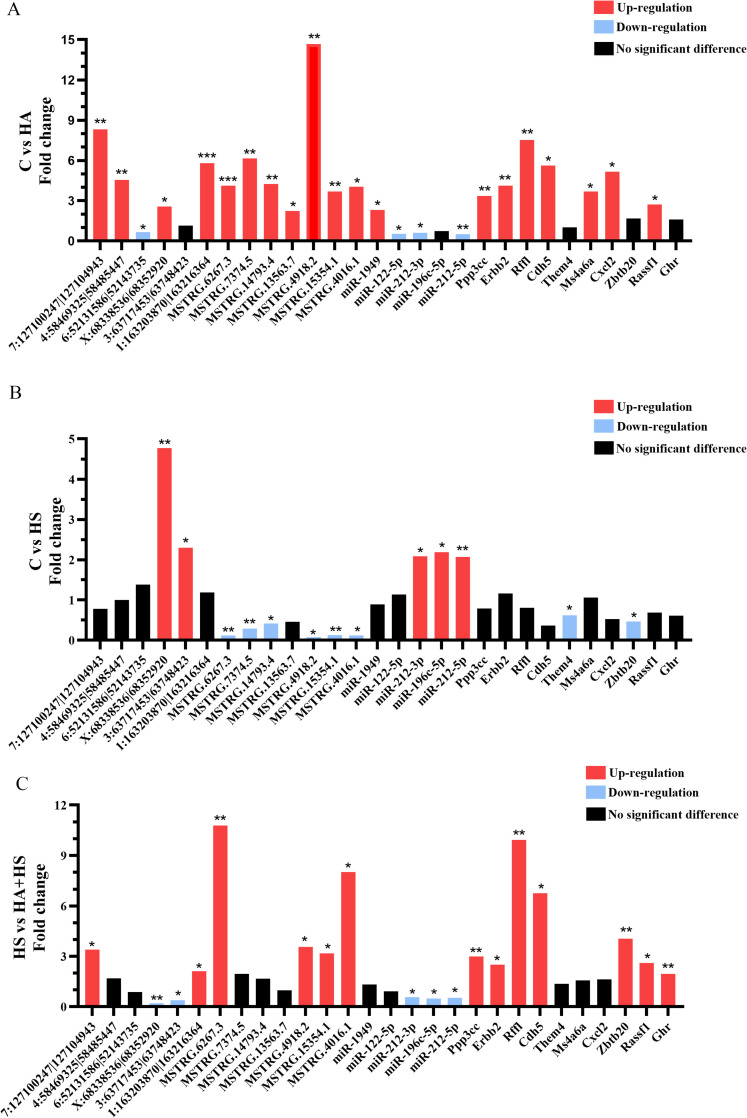
The expression of key transcripts was experimentally confirmed. Specifically, the fold change of 28 genes was analyzed in four different conditions: **(A)** C vs HA, **(B)** C vs HS, **(C)** HS vs HA + HS. * *P* < 0.05, ** *P* < 0.01, *** *P* < 0.001.

A total of 28 RNAs with significant disparities (6 circRNAS, 7 lncRNAs, 5 miRNAs and 10 mRNAs) were verified. The majority of these RNAs demonstrated significant differential expression and relatively high validation rates (Fig 4). In the comparison between Group C and HA, 7:127100247|127104943, MSTRG. 14793.4, rno-miR-122-5p, *Ppp3cc, Erbb2, Rffl* and *Cdh5* exhibited contradictory results, whereas the rest were consistent with the RNA-sequencing data. In the comparison between Group C and HS, *Zbtb20* exhibited contradictory results. When comparing Group HS to HA + HS, MSTRG.15354.1, *Ghr,* and *Erbb2* exhibited contradictory results versus the sequencing data.

### 3.6. Construction of the core circRNA\lncRNA-miRNA-mRNA network and functional analysis

**Fig 5:**
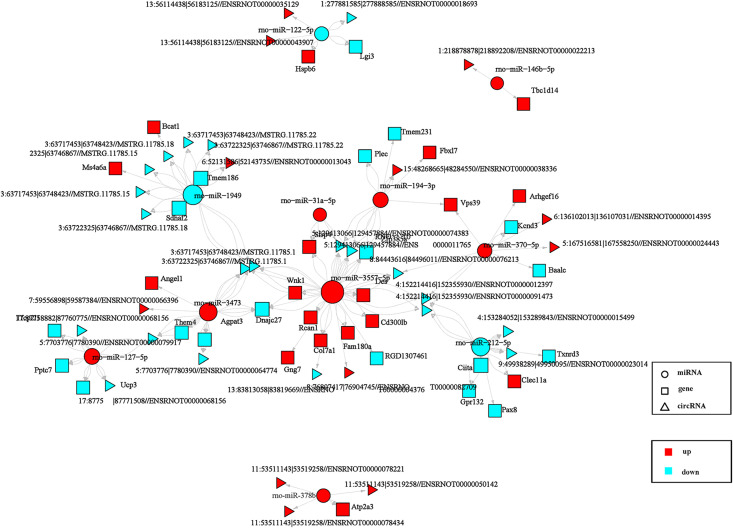
CircRNA-associated ceRNA networks. The network is composed of square nodes representing mRNAs, triangle nodes indicating lncRNAs or circRNAs, and circular frames denoting miRNAs. The Pearson correlation coefficient was utilized to establish the network, with an absolute value of PCC ≥ 0.50 and *P* < 0.05. The edges in the network represent the competing interactions among the nodes, while the colors red and blue indicate upregulated and downregulated expression, respectively.

**Fig 6:**
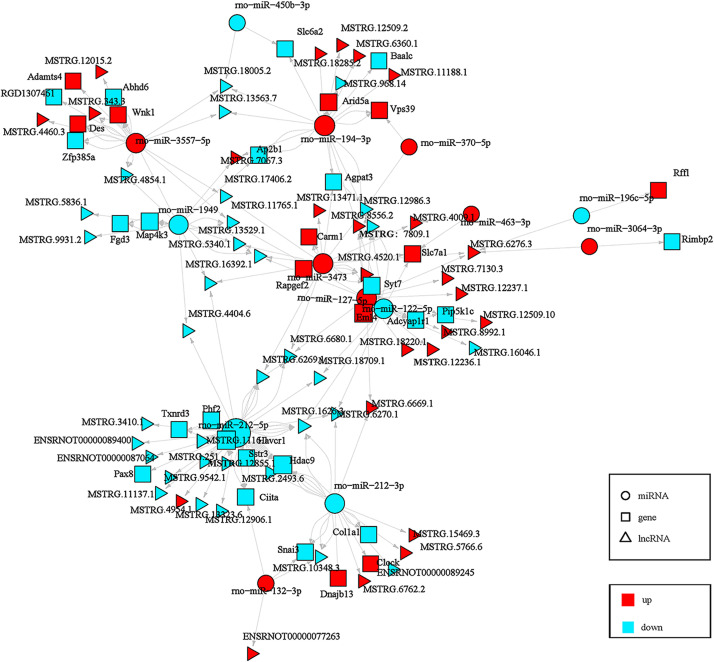
LncRNA-associated ceRNA networks. The presentation method is the same as that in Fig 5.

The ceRNA network was constructed by integrating based on the DEmiRNA–DEmRNA and DElncRNA/DEcircRNA–DEmiRNA regulatory network. Subsequently, an analysis of miRNA and mRNA functions from the literature was conducted to identify potential miRNAs and mRNAs associated with HA/HS. The resulting circRNA-related ceRNA network (31 DEcircRNAs, 11 DEmiRNAs, and 37 DEmRNAs) is presented in Fig 5, and the lncRNA-related ceRNA network (58 DElncRNAs, 14 DEmiRNAs, and 34 DEmRNAs) is shown in Fig 6. Based on the co-dysregulated ceRNA network of a candidate, both mRNAs and circRNAs/lncRNAs involved in this ceRNA crosstalk were negatively targeted and co-expressed with a common miRNA. In conjunction with the validated results of differentially expressed genes using RT-qPCR, two lncRNA-miRNA-mRNA pathways, namely MSTRG.6276.3-rno-miR-196c-5p-*Rffl* and MSTRG.4016.1-rno-miR-122-5p-*Rassf1* were identified.

**Fig 7:**
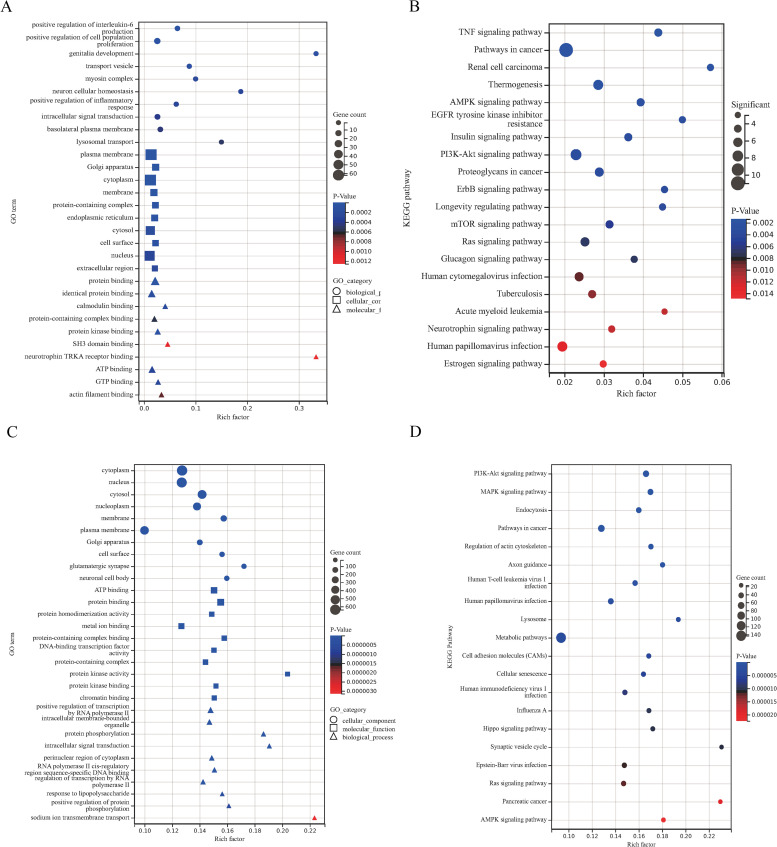
Top ten GO enrichment of circRNA\lncRNA-miRNA-mRNA regulatory network and analysis of related KEGG pathways. **(A)** CircRNA-associated-ceRNA network GO enrichment analysis bubble diagram. The x-axis represents the Rich factor, defined as the ratio of the number of differentially expressed genes annotated to a GO term to the total number of genes annotated to that term; a higher Rich factor indicates stronger enrichment. The y-axis shows −log₁₀(P value). The top 10 GO terms with the highest Rich factor are displayed. **(B)** CircRNA-associated-ceRNA network KEGG pathways analysis, bubble diagram, KEGG pathway enrichment bubble plot was generated using the same approach, with the top 20 KEGG pathways presented. Permission has been granted by the Kanehisa Laboratory (www.kanehisa,jp) [25, 26]. **(C)** LncRNA-associated-ceRNA network GO enrichment analysis bubble diagram. **(D)** LncRNA-associated-ceRNA network KEGG pathways analysis bubble diagram [25].

GO and KEGG pathway analyses of mRNAs embedded within the ceRNA networks delineate their functional specialization in HA-mediated protection (Fig 7). The circRNA-centric network is functionally linked to immunomodulatory and metabolic processes, including interleukin-6 production and cell population proliferation. This network operates primarily at the plasma membrane, Golgi, and cytoplasm, and is significantly enriched in key signaling pathways such as TNF, AMPK, and PI3K-Akt, suggesting a role in integrating inflammatory and energetic signals. In parallel, the lncRNA-centric network is associated with transcriptional regulation and intracellular signaling, including RNA polymerase II-driven transcription and protein phosphorylation. Its components localize to the nucleus and cytoplasm, and it prominently features the PI3K-Akt and MAPK signaling pathways, alongside endocytosis, implicating it in growth factor signaling and receptor dynamics.

The ceRNA networks—orchestrated by distinct circRNA and lncRNA cohorts—converge on a core set of signaling pathways, most notably the PI3K-Akt axis, to coordinate the adaptive cardiac response. These networks functionally segregate into immunometabolic regulation (circRNA-driven) and transcriptional/kinase signaling (lncRNA-driven), collectively fine-tuning critical processes that establish protection against heat stress.

## 4. Discussion

While heat acclimation (HA) confers robust protection against heat stress (HS)-induced injury, the comprehensive transcriptomic architecture—particularly the role of competing endogenous RNA (ceRNA) networks—underlying this cardioprotection remains poorly defined. ceRNA-mediated regulatory mechanisms have emerged as critical post-transcriptional modulators of the HS response across species, including dairy cattle [27], mice [28], and cellular models [15, 29], influencing processes from lactation physiology to systemic stress adaptation [30, 31]. Previous bioinformatic studies have described ceRNA expression changes in tissues such as liver [17], testis [28], hypothalamus [31] and pituitary [32] under thermal stress. However, the functional significance and network interactions of ceRNAs in the myocardium—specifically their role in mediating HA-induced protection—represent a gap. This study therefore employs whole-transcriptome sequencing of rat myocardium to systematically decode the ceRNA landscape, aiming to elucidate the regulatory logic by which non-coding RNA networks orchestrate cardiac resilience to heat stress.

### 4.1. HA protection against HS by regulation of lipid peroxidation and the inflammatory response

This study has demonstrated that HA has a significant impact on growth performance and thermoregulation in rats. Specifically, HA has been found to reduce growth performance, increase the core temperature, and decrease metabolic rate in rats. Furthermore, HA can affect energy and water metabolism, resulting in changes in appetite [33]. Studies have indicated that HS may lead to a decrease in body fat and protein production [34], which results in less weight gain compared with that in normal control rats. The study revealed that during HA, the Tre of rats exhibited a significant initial increase followed by a gradual decrease and stabilization, which is consistent with findings in previous research [22, 23]. A stable state of HA was achieved in rats housed at 35°C for 30 days. In our rat model of HS, Tre exceeded 40.4°C within three days of heat exposure, surpassing the core temperature observed in moderate HS conditions [11], indicating the successful establishment of the rat model of HS. Subsequently, our findings revealed that preconditioning with HA followed by HS exposure led to a decrease in Tre and the rate of weight loss, corroborating the results reported by Gupta et al [11] and indicating the successful establishment of the HA/HS model.

The present study reveals that HA can modulate oxidative stress and inflammatory response induced by HS. HSP70, a crucial regulatory factor in the process of HA, is known to exert anti-apoptotic effects and enhance the response of organism to anti-HS [35]. Our findings demonstrated that HSP70 expression was significantly upregulated in response to HS, which is consistent with previous reports [35, 36]. Notably, HSP70 expression is relatively low under normal conditions. These findings indicated a modest elevation in serum HSP70 levels following HA, which was further augmented in response to HS as a protective measure against HS-induced injury. Notably, the protective effect was enhanced in the context of HA and subsequent reheat stress.

HS is a physiological stress state induced by elevated ambient temperatures, which triggers systemic oxidative stress, impairs immune function, and elicits an inflammatory response, these factors collectively contribute to myocardial injury [37–39]. Lipid peroxidation, a key hallmark of oxidative damage, directly compromises cardiomyocyte membrane integrity and contractile function, whereas systemic inflammation exacerbates cardiac dysfunction and promotes adverse remodeling through the release of pro-inflammatory cytokines [40,41].The findings of this study indicate that exposure to HS resulted in an increase in serum MDA production, while simultaneously decreasing the levels of the anti-oxidative markers SOD and GSH-px, indicating the induction of systemic oxidative stress. However, pretreatment with HA could significantly reduce MDA and GSH-px levels compared with HS exposure alone. These results suggested that HA may maintains redox homeostasis to attenuate oxidative stress and protect cardiac function. Similarly, the balance between pro-inflammatory and anti-inflammatory mechanisms is critical for maintaining cardiac homeostasis. Specifically, these results showed that HS led to elevated IL-1β levels; however, this effect was mitigated upon HA reheat stress. The reduction in IL-1β suggests that HA helps limit the inflammatory response, thereby mitigating inflammation-driven myocardial injury. In the present study, a reduction in the pro-inflammatory marker IL-1β was observed, suggesting that HA preconditioning may aid in mitigating the inflammatory factors in the serum. Studies have shown that short-term HA increases plasma IL-6 levels in rats [39], whereas HS elevates serum IL-6 in humans [42]. In Tharparkar cattle, IL-6 expression rose under short-term HS but returned to baseline after long-term HA, indicating enhanced thermotolerance and modulation of inflammatory cytokine expression [43]. Similarly, IL-6 levels decreased in experimental animals following seven days of hot-water immersion, suggesting that HA mitigates HS-induced inflammation [44]. In these studies, HS preceded HA, with IL-6 levels declining after HA. In the present study, IL-6 levels were significantly elevated in the HS group, whereas they remained higher in the HA‑preconditioned group subsequently exposed to HS than in the HA group alone. This finding is consistent with previous observations that long-term HA restores IL-6 to baseline levels, whereas a more severe HS challenge can drive its elevation again. The comparable IL-6 levels between the long‑term HA group and the Con group further support the notion that IL-6 responds sensitively to HS intensity, likely reflecting a real‑time adaptive response to thermal stress. The staining results of rat myocardial tissue further corroborated this conclusion, indicating that HA can effectively safeguard myocardial tissue from inflammatory damage mediated by HS.

In summary, these findings suggest that HA may confer protection against HS-induced myocardial injury through multiple mechanisms, including upregulating HSP70 to enhance stress tolerance, mitigating systemic oxidative stress, and modulating inflammatory cytokine profiles. Although the degree of stress and the corresponding physiological responses may vary depending on the HA and HS protocols used, the overall pattern supports a protective role for HA in preserving cardiac function under thermal stress.

### 4.2. Functions of DERNA and regulation of bioinformatic pathways

The genes linked to the MHC protein, neuron, and axon may serve as crucial regulatory components in conferring protection against HS through HA. Additionally, pathways such as carbohydrate metabolism, immune system, transport and catabolism were deemed as significant heat regulation–related pathways. Furthermore, noteworthy findings were observed in the comparisons between the C and HS groups and the HS and HA + HS groups, where DEmRNAs were co-enriched in pathways related to phagosomes, cell adhesion molecules, calcium signaling, T helper (Th)17 cell differentiation, Th1 and Th2 cell differentiation, and viral myocarditis. These genes warrant further investigation in subsequent studies. Based on functional and pathway analyses of various groups, our findings suggest that the plasma membrane, synapses, mitochondria, and MHC protein may play crucial roles in heat regulation-related functions. Pathways related to the regulation of transport and catabolism, signal transduction, endocrine and metabolic processes, immune system, and environmental adaptation may also be important in heat regulation.

Using validation analysis, several crucial ceRNAs associated with heat regulation were identified. Previous research has demonstrated a 50% reduction in miR-196c-5p expression following a 10-day period of hypoxia acclimatization [45], suggesting that this microRNA is highly sensitive to hypoxic stress. Considering that elevated ambient temperatures can lead to tissue hypoxia, the current study explored the potential role of HA in facilitating cross-tolerance through the modulation of miR-196c-5p. The findings revealed a significant decrease in miR-196c-5p expression in the myocardium of rats subjected to HA preconditioning followed by HS, in comparison to those exposed to HS alone without prior adaptation. These findings suggest that HA may replicate essential molecular characteristics of hypoxia acclimatization by altering miR-196c-5p expression, thereby promoting cross-tolerance to hypoxic stress. MiR-196c-5p may serve as a critical molecular hub linking HA and hypoxia. HA-induced downregulation of miR-196c-5p likely enhances myocardial tolerance to subsequent HS—which is accompanied by tissue hypoxia—potentially through activation of HIF-related pathways. Thus, HA not only directly protects against HS-induced myocardial injury but also primes the heart to more effectively cope with hypoxic conditions by pre-engaging hypoxia-adaptive molecular programs, thereby exerting cardioprotective effects through cross-tolerance. Research has demonstrated that miR-212-3p plays a critical role in myocardial infarction injury by modulating apoptosis within cardiac tissue and is involved in cardiac hypertrophy via the regulation of autophagy-dependent apoptosis [46–48]. Furthermore, this miRNA inhibits inflammatory responses in macrophages [49] and is implicated in mediating ferroptosis and inflammation in sepsis-associated acute lung injury [50]. Notably, ferroptosis associated with sepsis is a significant pathway in HS-induced injury [51]. It was observed that miR-212-3p levels were markedly reduced in the myocardium of rats subjected to HA preconditioning compared to those exposed solely to HS. This finding suggests that after HA, miR-212-3p may alleviate HS-induced myocardial injury by attenuating excessive inflammation, regulating autophagy, or disrupting ferroptosis. Collectively, miR-212-3p appears to perform essential cardioprotective functions during HA through the integrated modulation of multiple pathways. MiR-212-5p has been implicated in various biological processes, including the suppression of nasopharyngeal carcinoma cell proliferation and the promotion of apoptosis [52], as well as the inhibition of neuroinflammation and enhancement of cognitive function [53]. In the present study, the expression of miR-212-5p was significantly reduced in the myocardium of heat-acclimated rats compared to non-acclimated rats subjected to HS. Given its established roles in regulating inflammation, proliferation, and apoptosis, miR-212-5p may contribute to HA-induced cardioprotection through the modulation of inflammatory responses and apoptosis. Additionally, miR-1949 has been associated with apoptosis [54]. The findings indicated that miR-1949 expression was elevated following HA but did not change significantly after HS alone, suggesting that this miRNA may be involved in myocardial protective adaptation during the establishment phase of HA. Therefore, miR-1949 likely plays a role primarily in the early stages of HA rather than in the direct response to acute HS, although its precise mechanisms require further investigation.

*Rassf1* promotes apoptosis of cardiomyocytes following acute myocardial infarction [55]. *Rassf1* expression was elevated in the myocardium of heat-acclimated rats compared with C rats, as well as in the myocardium of HA preconditioned HS rats compared with HS rats. Bioinformatics analysis revealed that *Rassf1* is significantly enriched in the Hippo and Ras signaling pathways, both of which have been implicated in response to HS and HA [23, 56, 57]. Therefore, it is plausible that *Rassf1* may be involved in cell apoptosis and that it plays a pivotal role in the protection of HA against HS. *Rffl* participates in multiple biological processes by facilitating ubiquitin-mediated proteasomal degradation, with potential roles in endocytic recycling [58]and damaged mitochondria regulation [59, 60]. This study found that Rffl was highly expressed in the myocardium of HA rats and HA-preconditioned rats subjected to HS, whereas HS alone had no significant effect. Furthermore, Enrichment analysis indicated that *Rffl* is involved in neuroactive ligand-receptor interaction, cytokine-cytokine receptor interaction, and PI3K-Akt signaling pathways. Therefore, *Rffl* may confer protection against HS-induced myocardial injury during HA by modulating endocytic recycling and mitochondrial pathways. *Zbtb20* plays a crucial role in various biological processes, such as neurogenesis, and glucose and lipid metabolism homeostasis [61–63]. Previous research shows that *Zbtb20* is highly expressed in the hypothalamus of rats following HA [23]. This finding revealed that *Zbtb20* expression in the myocardium of rats decreased in response to HS, but increased following HA preconditioning. Furthermore, *Zbtb20* is enriched in the Hippo and Ras signaling pathways, suggesting that it may be involved in glucose and lipid metabolism homeostasis and have a protective function against HS injury via these pathways. *Ppp3cc p*lays a crucial role in intracellular Ca^2+^-mediated signals transduction, participates in the downstream regulation of dopaminergic signal, and has been associated with heat tolerance in Dehong hump cattle [64]. In this study, *Ppp3cc* expression increases following exposure to HA and HA preconditioned HS, but is not influenced by HS alone. Enrichment analysis revealed its involvement in the calcium signaling pathway, suggesting that *Ppp3cc* may enhance heat tolerance via this pathway and may be involved in HA-mediated protection against HS-induced myocardial injury. *Cxcl2* is generated by activated monocytes and neutrophils and is expressed at sites of inflammation [65], and enhances the transcriptional activity of HIF-1α [66]. HA was found to upregulate *Cxcl2* expression, thereby implying that it may regulate inflammation and HIF-1α to promote HA. *Cdh5* represents a class of calcium-dependent cell adhesion proteins that potentially interacts with HIF-2α [67], and plays a critical role in the immune response [68]. HA upregulated *Cdh5* expression, suggesting that it may contribute to HA-mediated protection against HS by modulating HIF-2α and inflammatory responses. *Erbb2* overexpression can inhibit stress-induced autophagy [69]. The signaling pathway mediated by ERBB2 is indispensable in maintaining normal cardiac function and responding to cellular stressors [70], and may regulates ferroptosis [71]. *Erbb2* expression was found to be upregulated following exposure to HA and HA preconditioned HS, suggesting that *Erbb2* may play a role in regulating autophagy and ferroptosis to mitigate HS-induced damage.

Collectively, HA orchestrates a coordinated myocardial protective network by modulating key gene targets—including specific miRNAs and mRNAs—that regulate apoptosis, autophagy, ferroptosis, metabolism, calcium signaling, inflammation, and hypoxia responses. Through these targets, HA confers effective protection against HS-induced myocardial injury. These molecules may also serve as potential biomarkers for HA/HS, although their precise mechanisms warrant further investigation.

### 4.3. Two lncRNA-miRNA-mRNA networks in the regulation HA can protect against HS

This study constructed ceRNA networks with DEmiRNAs as central regulators, DEmRNAs as functional targets, and DEcircRNAs/DElncRNAs as competing molecular decoys. Through this approach, two key regulatory axes were identified: MSTRG.6276.3-miR-196c-5p-*Rffl* and MSTRG.4016.1-miR-122-5p-*Rassf1*. Functional analysis suggests the MSTRG.6276.3-miR-196c-5p-*Rffl* axis may mediate cellular adaptation through regulation of hypoxia response pathways, endocytic recycling, and mitophagy. Meanwhile, the MSTRG.4016.1-miR-122-5p-*Rassf1* axis appears to modulate inflammatory responses and cellular homeostasis via the Hippo and Ras signaling pathways. These findings reveal a sophisticated ceRNA network architecture wherein specific lncRNAs function as molecular sponges to sequester miRNAs and derepress protective gene expression programs. Collectively, these axes may represent critical components through which HA confers protection against HS-induced myocardial injury. Although this study provides fundamental insights into the non-coding RNA landscape underlying HA-induced cardioprotection, the precise mechanistic contributions of these regulatory axes warrant further experimental validation.

## 5. Conclusions

The current investigation offers a methodical portrayal of the expression profiles of the rat myocardial transcriptome in the context of HA protection against HS. HA preconditioning significantly attenuates HS-induced elevation of core temperature and alleviates oxidative stress and inflammation by preserving redox homeostasis, reducing the pro-inflammatory cytokine IL-1β, and dynamically regulating IL-6 expression. HA also modulates multiple functional genes, including *Rffl, Zbtb20, Ppp3cc, Cdh5, Erbb2*, and *Cxcl2*, which may be involved in maintaining glycolipid metabolism homeostasis, regulating inflammation, autophagy, and ferroptosis, collectively forming a regulatory network that counteracts HS-induced myocardial injury. At the non-coding RNA level, HA downregulates miR-196c-5p, miR-212-3p, and miR-212-5p, upregulates miR-1949, and establishes two ceRNA regulatory axes, MSTRG.6276.3-miR-196c-5p-*Rffl* and MSTRG.4016.1-miR-122-5p-*Rassf1*, which are involved in hypoxia response, cellular homeostasis, and inflammatory regulation. These findings indicate that HA confers myocardial protection through a multi-level, multi-target synergistic mechanism, providing a systematic transcriptomic basis for understanding the regulatory network underlying HA-mediated protection against HS-induced myocardial injury.

## Abbreviations

HA: Heat acclimation
HS: Heat stress
RH: Relative humidity
C: Control
*Rffl*: ring finger and FYVE-like domain containing E3 ubiquitin protein ligase
*Rassfl*: Ras association domain family member 1
Ghr: Growth hormone receptor
*Zbtb20*: zinc finger and BTB domain containing 20
Them4: Thioesterase uperfamily member 4
*Chd5*: cadherin 5
*Erbb2*: erb-b2 receptor tyrosine kinase 2
*Cxcl2*: C-X-C motif chemokine ligand 2
*Ppp3cc*: protein phosphatase 3 catalytic subunit gamma
Ms4a6a: membrane spanning 4-domains A6A
HSP70: Heat shock protein 70
MDA: Malondialdehyde
SOD: Superoxide dismutase
GSH-px: Glutathione peroxidase
IL-1β: Interleukin-1β
IL-4: Interleukin-4
IL-6: Interleukin-6
GO: Gene ontology
KEGG: Kyoto Encyclopedia of Genes and Genomes
ADG: Average daily gain
ADFI: Average daily feed intake
F/G: ratio of feed to gain
DEmRNAs: Differentially expressed mRNAs
DEmiRNAs: Differentially expressed miRNAs
DElncRNAs: Differentially expressed lncRNAs
DEcircRNAs: Differentially expressed circRNAs
